# Naringenin Suppresses the Hyperexcitability of Trigeminal Nociceptive Neurons Associated with Inflammatory Hyperalgesia: Replacement of NSAIDs with Phytochemicals

**DOI:** 10.3390/nu16223895

**Published:** 2024-11-15

**Authors:** Sora Yajima, Risa Sakata, Yui Watanuki, Yukito Sashide, Mamoru Takeda

**Affiliations:** Laboratory of Food and Physiological Sciences, Department of Life and Food Sciences, School of Life and Environmental Sciences, Azabu University, 1-17-71, Fuchinobe, Chuo-ku, Sagamihara 252-5201, Kanagawa, Japan; f21006@azabu-u.ac.jp (S.Y.); tenis.risa0711@gmail.com (R.S.); v21065@azabu-u.ac.jp (Y.W.); me2401@azabu-u.ac.jp (Y.S.)

**Keywords:** inflammation, trigeminal nociceptive neuron, hyperalgesia, pathological pain, extracellular single-unit recording, diclofenac, NSAIDs, naringenin, phytochemical, complementary alternative medicine

## Abstract

The present study examines whether the systemic application of naringenin (NRG) reduces inflammation-induced hyperexcitability in the spinal trigeminal nucleus caudalis (SpVc) related to hyperalgesia, and compares its impact with that of diclofenac (DIC). To provoke inflammation, the whisker pads of rats were injected with complete Freund’s adjuvant, and subsequently, mechanical stimuli were administered to the orofacial region to determine the escape threshold. Compared to naïve rats, the inflamed rats showed a significantly lower mechanical threshold, and this reduced threshold returned to normal levels two days post-administration of NRG, DIC, and half-dose DIC plus half-dose NRG (1/2 DIC + 1/2 NRG). Using extracellular single-unit recordings, the activity of SpVc wide-dynamic range neurons was measured in response to mechanical stimulation of the orofacial area under anesthesia. The average firing rate of SpVc neurons when exposed to both non-painful and painful mechanical stimuli was significantly reduced in inflamed rats following NRG, DIC, and 1/2 DIC + 1/2 NRG administration. The heightened average spontaneous activity of SpVc neurons in rats with inflammation was significantly reduced following NRG, DIC, and 1/2 DIC + 1/2 NRG administration. The increased average receptive field size observed in inflamed rats reverted to normal levels after either NRG, DIC, or 1/2 DIC + 1/2 NRG treatment. These findings indicate that NRG administration can reduce inflammatory hyperalgesia linked to the heightened excitability of SpVc wide-dynamic range neurons.

## 1. Introduction

Painful sensory signals originating from the orofacial region are transmitted through small Aδ-fibers and unmyelinated C-fibers of trigeminal ganglion (TG) neurons to second-order neurons in the spinal trigeminal nucleus caudalis (SpVc) [1,2,3]. Neurons in the SpVc that process nociceptive signals are classified as either nociceptive-specific or wide-dynamic range (WDR) based on how they react to mechanical stimulation in orofacial regions, such as the facial skin. Both non-noxious and noxious stimuli activate SpVc WDR neurons [3]. The application of noxious stimuli with varying intensity to the receptive fields results in SpVc WDR neurons firing at higher frequencies in accordance with the strength of the stimulus, suggesting that WDR neurons play a crucial role in encoding stimulus intensity. Rat models of orofacial inflammation induced by complete Freund’s adjuvant (CFA) have been established to investigate the trigeminal neural pathways associated with pathological pain, where increased responsiveness of SpVc WDR neurons in response to mechanical stimuli is observed [4,5]. SpVc WDR neurons have also been suggested to play a role in causing hyperalgesia and/or referred pain related to dental pain [2,3,4,5].

In Western countries, lately, there has been a rise in the utilization of complementary and alternative medicine (CAM) for patients who do not respond to Western medical treatments like drug therapy, with a focus on its potential for managing chronic pain [6], suggesting the capability of complementary and alternative medicines (CAMs) to prevent trigeminal inflammatory hyperalgesia. Prior research has indicated that the long-term intake of dietary components, such as polyphenols and carotenoids, can reduce inflammation-associated pain sensitivity by dampening the hyperactivity of SpVc WDR neurons through inhibition of both peripheral and central cyclooxygenase-2 (Cox-2) cascade signaling pathways [7,8]. These findings indicate that natural products derived from fish, vegetables, and fruits contribute to analgesia, including that related to inflammatory pain.

Naringenin (NRG; 4′,5,7-thrihydrooxyflavanone) is one of the prevalent types of flavonoids; it is a plant metabolite that acts as a dietary phytochemical commonly found in the human diet [9]. Previous studies reported that daily treatment with NRG reduced CFA-induced mechanical hyperalgesia without any side effects [10]. NRG performs multiple biological roles, including antioxidant and anti-inflammatory, and it also exhibits minimal toxicity, as a result, it holds the possibility of being employed as a therapy tool [11,12]. Concerning excitable tissues, a modulatory role has been noted in reports for NRG on transient receptor potential melastatin 3 (TRPM3) and voltage-gated Na (Nav) and Ca^2+^ (Cav) channels in sensory neurons [13,14]. For example, Zhou et al. [13] demonstrated that, under in vitro conditions, the application of NRG inhibits both Cav and Nav currents in the primary sensory neurons of the dorsal root ganglion (DRG). On the other hand, under inflammatory conditions, NRG induces analgesia through activation of the nitric oxide (NO)/cyclic guanosine monophosphate (c-GMP)/protein kinase G (PKG)/adenosine triphosphate (ATP)-sensitive K channel pathway, reducing neuronal pain transmission in inflammatory pain models [10,15]. In addition, NRG can also decrease the production of prostaglandin E_2_ (PGE_2_) by inhibiting Cox-2 cascades [16], suggesting that NRG might be a potential agent for inhibitory neuronal excitability associated with inflammatory hyperalgesia. To date, in vivo, the neurophysiological mechanism of how NRG suppresses the hyperexcitability of nociceptive neurons induced by inflammation has not been assessed using electrophysiological approaches.

While non-steroidal anti-inflammatory drugs (NSAIDs) are powerful and effective in inhibiting Cox-2 for pain relief, they unfortunately come with unwanted side effects (e.g., stomach ulcers and heart attacks) due to their pharmacological properties [17,18]. Diclofenac (DIC) is a well-established and frequently prescribed NSAID known for its pain-relieving, anti-inflammatory, and fever-reducing effects and has proven effective in managing a range of both acute and chronic pain and inflammatory conditions [19]. To date, there have been reports of comparative analyses of the analgesic effects of phytochemicals and NSAIDs on hyperalgesia [9], but there have been no studies that have examined the effect of substituting a part of the DIC dose with phytochemicals. Therefore, using behavioral and electrophysiological techniques, the present study investigates whether NRG administration under in vivo conditions attenuates inflammation-induced hyperexcitability of the SpVc neurons associated with hyperalgesia in rats. In addition, we examine and compare the potency of suppression of hyperalgesia-associated, inflammation-induced, SpVc neuronal excitability with NRG and NSAIDs, such as DIC. Finally, we also examine whether the systemic injection of a half-dose of the conventional NSAID, DIC, replaced by a half-dose of NRG, could contribute to CAM therapies without any side effects. As a result, we found that administration of NRG attenuated inflammatory hyperalgesia associated with hyperexcitability of SpVc WDR neurons. We found evidence that the magnitude of NRG-mediated inhibition on the hyperexcitability of SpVc neurons associated with hyperalgesia was almost equal to 1/2 DIC + 1/2 NRG administration. These findings support the potential of NRG as a therapeutic agent in CAM strategies for preventing trigeminal inflammatory mechanical hyperalgesia. Taken together, our findings also suggest the possibility of treating pain in a natural way through the intake of certain dietary and nutritional ingredients that contain naringenin.

## 2. Materials and Methods

The Animal Use and Care Committee of Azabu University authorized all the experiments mentioned in this document (No. 230120-11). All experiments complied with the ethical rules of the International Association for the Study of Pain [20]. Maximum efforts were taken to reduce the number of animals used and to alleviate their suffering. Each experiment was performed by researchers, and experimenters were blinded to the experimental conditions of the study.

### 2.1. Triggering Inflammation and Applying NRG Alongside NSAIDs

Adult male Wistar rats, each weighing 215 to 245 g, were maintained under stable illumination settings (lights are turned on from 7:00 a.m. to 7:00 p.m.). While it has been noted that there are sex differences in reactions to experimental pain, the precise mechanisms responsible for these differences are not yet fully understood [21]. Thus, this study was conducted using only male rats. The experiments were performed on adult male Wistar rats (n = 30). The rats were categorized into five different groups, with the following classifications: (i) naïve (n = 6); (ii) inflamed (n = 6); (iii) inflamed rats with NRG (50 mg/kg, i.p.; Sigma-Aldrich, Milano, Italy) treatment (n = 6); (iv) inflamed rats with DIC (50 mg/kg, i.p.; Sigma-Aldrich) treatment (n = 6); (v) inflamed rats with DIC (25 mg/kg, i.p.) + NRG (25 mg/kg, i.p.) treatment (n = 6). Previous studies indicated that these doses of NRG and DIC significantly suppress Cox-2 activity in vitro [10,16,19]. Each animal underwent anesthesia with 3% isoflurane, followed by the injection of 0.05 mL CFA (a 1:1 oil/saline mixture) into the left facial skin, as described previously [7,8]. Naïve rats received an injection of only the vehicle solution (0.9% NaCl) on the left side of their facial skin. Over a period of two days, NRG and DIC were solubilized in dimethyl sulfoxide (DMSO) and continuously administered to the rats. Behavioral experiments were carried out just before the daily administration. In this research, after assessing the escape threshold behavior, electrophysiological experiments were carried out exclusively on the second day in rats from the naïve, CFA, CFA-inflamed + NRG, CFA-inflamed + DIC, and 1/2 NRG + 1/2 DIC groups. Once the electrophysiological experiments were concluded, the rats were euthanized right away using an overdose of the anesthetic agent pentobarbital sodium (200 mg/kg, i.v.).

### 2.2. Threshold for Triggering Escape Behavior

The method used to identify the mechanical threshold for escape behavior was conducted as previously described [7,8]. In summary, the examination for mechanical hyperalgesia was conducted on the skin areas on both the ipsilateral and contralateral sides utilizing a set of von Frey hairs (Semmes-Weinstein Monofilaments, North Coast Medical, Morgan Hill, CA, USA) one to two days after the CFA or vehicle was injected into the facial skin. To assess the rat’s escape threshold, the whisker pad was subjected to von Frey mechanical stimuli applied in an ascending order of trials using an inter-stimulus interval of 5 s. Each von Frey stimulus was administered three times per series of trials with a 5-s interval. The escape threshold intensity was identified when the rats turned their heads away from at least one of the three presented stimuli. In this study, we determined inflammatory hyperalgesia in behavioral experiments by a statistically significant decrease in the withdrawal threshold in the CFA-inflamed group compared to the naive group.

### 2.3. Recording of Neuronal Activity in SpVc WDR Neurons Using Extracellular Single-Unit Methods

Two days post-injection of CFA or vehicle, electrophysiological recordings were performed as previously described. Electrophysiological data were collected from 30 adult male Wistar rats. Each rat was sedated using 3% isoflurane and kept under anesthesia (0.3 mg/kg of medetomidine, 4.0 mg/kg of midazolam, and 5.0 mg/kg of butorphanol, i.p.) with supplementary doses of an anesthetic mixture at a rate of 2–3 mg/kg/h as needed, through a cannula into the jugular vein. During the recording session, the absence of response to paw pinching was used to confirm the depth of anesthesia. The rectal temperature was kept stable at 37.0 °C ± 0.5 °C using a homeothermic blanket (Temperature Controller 40-90-8D; FHC, Aspen, Tokyo, Japan) during recording. All wound margins were consistently treated with a local anesthetic solution, specifically 2% lidocaine (Xylocaine), throughout the course of the experiments. The animals were subsequently arranged in a stereotaxic instrument (SR-50; Narishige, Tokyo, Japan), and their neck muscles were split along the midline. The medullary brain stem was made accessible by creating an incision through the atlanto-occipital ligament and dura mater. Extracellular recording of single-unit activity from the SpVc area was achieved using a tungsten microelectrode (3–5 MΩ) inserted into the same side of the medulla, and a micromanipulator was used to adjust the position in 10 μm increments or decrements (SM-11 and MO-10; Narishige, Tokyo, Japan), according to the stereotaxic coordinates of the rat brain atlas of Paxinos and Watson [22]. Neural activity underwent amplification (DAM80; World Precision Instruments, Sarasota, FL, USA), filtration (0.3–10 KHz), monitoring with an oscilloscope (SS-7672; Iwatsu, Tokyo, Japan), and was recorded for subsequent analysis using Power Lab equipment Chart 5 software (ADInstruments, Oxford, UK), as described previously [7,8].

### 2.4. Neuronal Activity Measurements

The examination of extracellular single-unit SpVc WDR responses to mechanical stimulation of the whisker pad was performed in the following manner. To prevent the sensitization of peripheral mechanoreceptors, a paintbrush was swiftly employed as a probing tool to pinpoint the general location of the receptive field on the left side of the whisker pad. Subsequently, we investigated the left side of the whisker pad to find single units that reacted to a variety of von Frey hairs, including both non-noxious (0.2, 0.6, 2, 6, 10 g) and noxious (15, 26, 60 g) mechanical stimulation for 5 s at intervals of 5 s [7,8]. We established that the criterion for WDR neurons involved a graded response to mechanical stimulation applied to the receptive area; both non-noxious and noxious stimuli were used.

Following the identification of nociceptive SpVc WDR neurons that reacted to the whisker pad, we ascertained the mechanical stimulation threshold and documented the receptive field’s size. To locate the mechanical receptive zones of neurons, von Frey hairs were used on the facial skin, and these areas were subsequently traced onto a life-size drawing of the rat on tracing paper [7,8]. Quantification of WDR neuronal activity upon mechanical stimulation was achieved by deducting the background neuronal activity from the activity prompted by the stimulation. The frequencies of spontaneous discharges were assessed within a 2 to 5-min timeframe. In this study, we determined inflammatory hyperexcitability of SpVc WDR neurons, as following criteria: (i) a statistically significant increase in discharge frequency to mechanical stimulation; (ii) a decrease in the threshold to mechanical stimulation; (iii) an increase in spontaneous discharge frequency; and (iv) an increase in receptive field size in the CFA-inflamed compared to the naive group [8]. Previous research has revealed that (i) WDR neurons within the SpVc region are significantly involved in the mechanisms that cause mechanical hyperalgesia [3], and (ii) evidence suggests that nociceptive-specific neurons may convert to WDR neurons after CFA inflammation occurs [1,23]. As a result, this research focused on the effects of NRG on the nociceptive SpVc WDR neuronal activity, which means we did not explore nociceptive-specific neurons. In response to each stimulus, peristimulus histograms with 100 ms bins were created. The average spontaneous and mechanically induced discharge frequencies, along with the average mechanical thresholds, of SpVc WDR neurons were compared across the five animal groups (naïve, CFA, CFA with NRG treatment, CFA with DIC treatment, and CFA with 1/2 NRG + 1/2 DIC treatment). Using the micromanipulator, the single-unit recording positions within the SpVc region were determined and mapped, referencing their distance from the obex medial line and surface of the medullary dorsal horn, according to the rat brain atlas, as outlined in our prior studies [7,8].

### 2.5. Data Analysis

Values are expressed as means ± SEM. A one-way repeated measures analysis of variance was utilized for statistical analysis and subsequent Tukey–Kramer’s post hoc tests and Student’s *t*-test were performed on the behavioral and electrophysiological data (Excel Statcel 4). A *p*-value less than 0.05 was regarded as indicating a significant difference.

## 3. Results

### 3.1. Hyperalgesia Resulting from Inflammation

**Figure 1:**
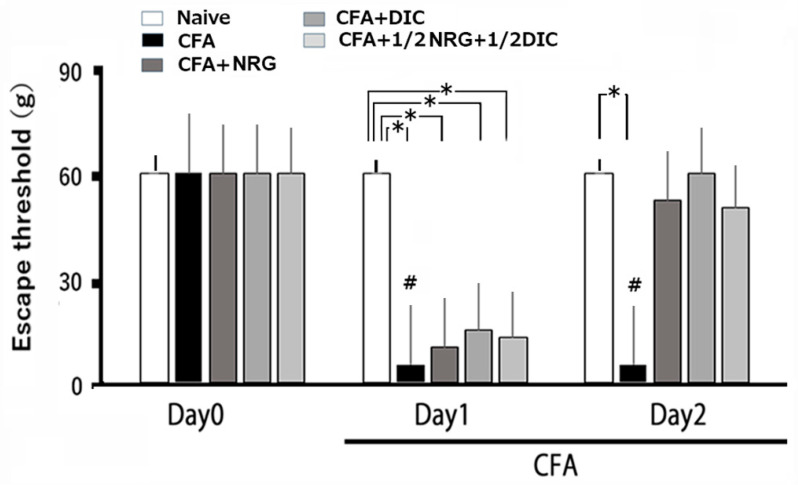
Analyzing variations in escape thresholds across different conditions: naïve, inflamed, inflamed treated with naringenin (NRG), inflamed treated with diclofenac (DIC), and inflamed with 1/2 DIC and 1/2 NRG rats. The ipsilateral whisker pad of rats was subjected to mechanical stimulation using von Frey filaments in five experimental groups: naïve (saline; n = 6), complete Freund’s adjuvant (CFA)-inflamed (n = 6), CFA-inflamed with NRG (50 mg/kg, i.p.; n = 6), DIC (50 mg/kg, i.p.; n = 6), or 1/2 DIC and 1/2 NRG groups to assess hyperalgesia. Data are mean ± SEM; ^#^ *p* < 0.05 when comparing inflamed day 0 vs. inflamed day 1, day 2; * *p* < 0.05.

After injecting CFA into the whisker pad, hyperalgesia in the rats was evaluated by probing the site of injection and/or the facial skin. As illustrated in Figure 1, CFA notably lowered the escape threshold in inflamed rats subjected to mechanical stimulation on the whisker pad region from 63.2 ± 2.9 g in naïve rats to 8.6 ± 2.5 g at day 2 after the injection (n = 6, *p* < 0.05; Figure 1). As described previously, there were no significant changes observed in the naïve rats after vehicle injection [7,8]. The contralateral threshold levels in the whisker pad region showed no marked variation between the groups. (naïve vs. inflamed; 62.3 ± 6.6 g vs. 65.4 ± 5.2 g, n = 6, not significant [NS]).

### 3.2. Chronic Administration of NRG, DIC, and 1/2 DIC + 1/2 NRG for Hyperalgesia

With daily NRG doses, the escape threshold from mechanical stimulation that was lowered in inflamed rats on day 1 was partly restored to normal (Figure 1). Figure 1 illustrates that the lowered escape threshold caused by mechanical stimulation in CFA-inflamed rats was restored to normal levels after administration (naïve vs. day 2 CFA-inflamed + NRG; 63.2 ± 2.9 g vs. 57.2 ± 6.7 g, n = 6, NS). Furthermore, the lowered escape threshold from mechanical stimulation in inflamed rats reverted to normal levels after administrating DIC on day 2 (naïve vs. day 2 inflamed with DIC; 63.2 ± 2.9 g vs. 59.2 ± 7.7 g, n = 6, NS). On day 2, no notable difference was observed between the inflamed rats treated with NRG and those treated with DIC. Moreover, the decreased escape response threshold from mechanical stimulation in inflamed rats was restored to normal levels after 1/2 DIC + 1/2 NRG at day 2 after inflammation (naïve vs. day 2 inflamed with 1/2 DIC + 1/2 NRG; 63.2 ± 2.9 g vs. 53.2 ± 6.5 g, n = 6, NS). No notable difference was observed on day 2 between naïve rats and inflamed rats treated with 1/2 DIC + 1/2 NRG.

### 3.3. Modifications in the Excitability of SpVc WDR Neurons After Inflammation

**Figure 2:**
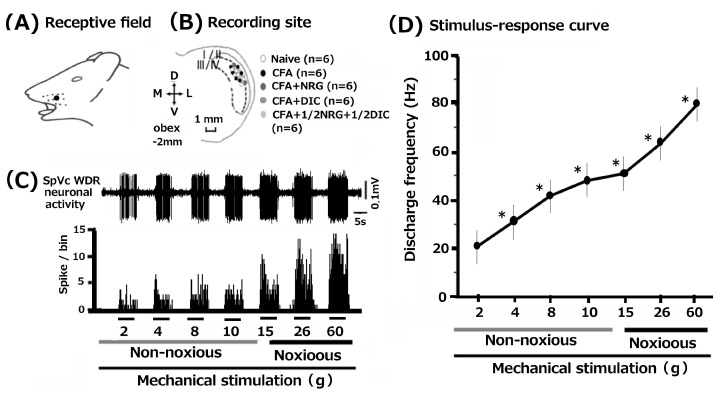
Common features of wide-dynamic range (WDR) neuronal activity in the spinal trigeminal nucleus caudalis (SpVc) responding to mechanical stimulation in orofacial skin. (**A**) The region of the facial skin corresponding to the whisker pad’s sensory field. (**B**) The distribution of SpVc WDR neurons responding to non-noxious and noxious mechanical stimulation of facial skin (n = 30). The configuration of SpVc WDR neurons involved in responding to mechanical non-noxious and noxious on the facial skin (n = 30). (**C**) An example of firing in SpVc WDR neurons in an untreated rat, triggered by both non-noxious and noxious mechanical stimulation. (**D**) The stimulus–response relationship for SpVc WDR neurons (n = 30). * *p* < 0.05 for comparison of 2 g vs. 6 g, 10 g, 15 g, 26 g, and 60 g.

In total, 30 SpVc WDR neurons were examined by mechanical stimulation of the whisker pad in rats among the naïve, inflamed, inflamed + NRG, inflamed + DIC, and 1/2 DIC + 1/2 NRG groups. Figure 2A illustrates that SpVc WDR neurons, responsive to both non-noxious and noxious mechanical stimuli, exhibited a somatic receptive field in the whisker pad (layers I-II, n = 6, 20%; layers III-V, n = 24, 80%) and they were typically distributed in the maxillary branch (Figure 2B). The recording sites appeared consistent among the five groups. Every SpVc neuron assessed demonstrated an increased firing rate when mechanical stimulation of varying intensities was applied to the most sensitive area within the receptive field (Figure 2D). In Figure 2D, the correlation between mechanical stimulus intensity and the mean firing frequency of WDR neurons is depicted as a stimulus–response graph. Thus, the analyzed neurons were all identified as WDR neurons (Figure 2C), as detailed in our prior study [7,8].

### 3.4. Changes in Excitability of SpVc WDR Neurons Following Inflammation

**Figure 3:**
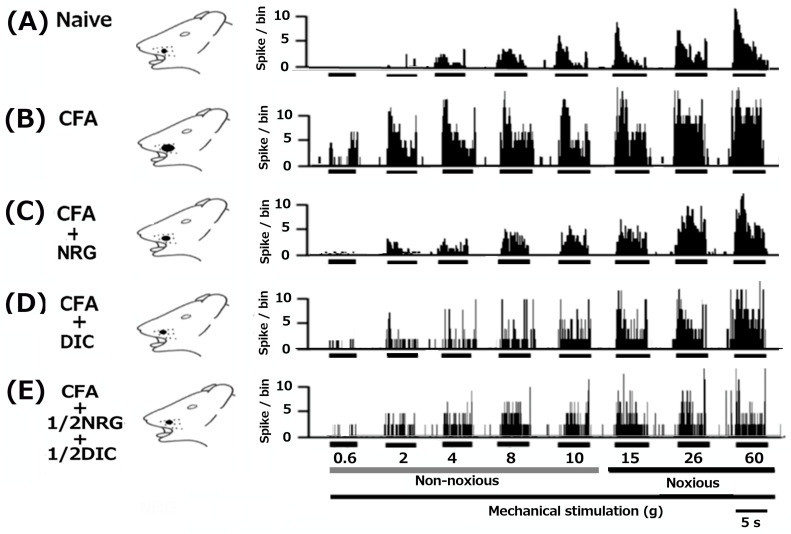
Following orofacial CFA inflammation, the hyper-excitability of SpVc WDR neuronal activity was reversed by the administration of chronic NRG, DIC, and a mix of 1/2 DIC and 1/2 NRG. Example showing the reaction of SpVc WDR neurons to non-noxious and noxious mechanical stimuli in subjects that are naïve (**A**), suffering from inflammation (**B**), and suffering from inflammation treated with NRG (50 mg/kg, i.p. for two days) (**C**), inflamed with DIC (50 mg/kg, i.p. for two days) (**D**) and inflamed with 1/2 DIC + 1/2 NRG (for two days) rats (**E**). It should be noted that a lower threshold for mechanical stimulation is needed to trigger neuronal activity, which also results in more frequent spontaneous nerve impulses and increased receptive field size in inflamed rats returned to control levels following NRG, DIC, 1/2 DIC + 1/2 NRG administration for two days.

**Figure 4:**
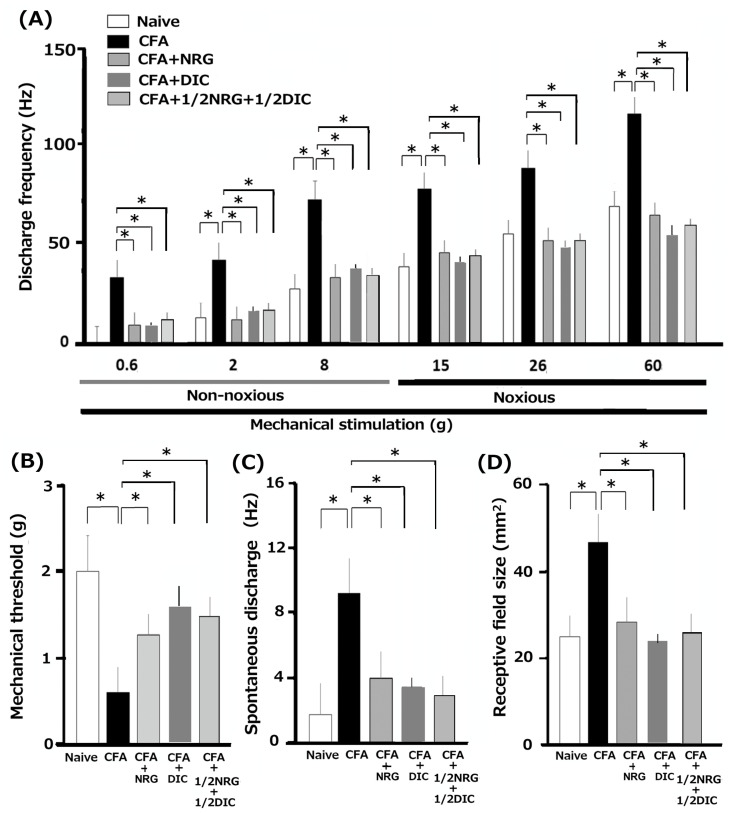
A summary of persistent NRG or DIC treatments, alongside a combination therapy of half DIC and half NRG, restored elevated SpVc WDR neuronal activity following orofacial inflammation induced by CFA inflammation. (**A**) Analysis of the average discharge rate of SpVc WDR neurons in response to both non-noxious and noxious mechanical stimulation of orofacial skin across the five groups (each group, n = 6). * *p* < 0.05 comparing naïve vs. inflamed rats and inflamed vs. inflamed with NRG or DIC and inflamed with 1/2 DIC + 1/2 NRG. (**B**) Evaluating the mean mechanical threshold of SpVc WDR neurons across the five groups of rats. * *p* < 0.05 comparing naïve vs. inflamed rats and inflamed vs. inflamed with NRG or DIC and inflamed with 1/2 DIC + 1/2 NRG. (**C**) Spontaneous activity of SpVc WDR neurons across the five groups of rats. * *p* < 0.05 comparing naïve vs. inflamed rats and inflamed vs. inflamed with NRG or DIC and inflamed with 1/2 DIC + 1/2 NRG. (**D**) An analysis of the differences in mean receptive field sizes of SpVc WDR neurons among the five rat groups. * *p* < 0.05 comparing naïve vs. inflamed rats and inflamed vs. inflamed with NRG or DIC and inflamed with 1/2 DIC + 1/2 NRG.

As mentioned in our prior studies, we initially verified that CFA causes hyperexcitability in SpVc WDR neurons [7,8]. In naïve rats, spontaneous discharges were observed in 16.7% (1/6) of SpVc neurons (Figure 3A and Figure 4C). Most neurons exhibited low-frequency firing, averaging 1.5 ± 2.1 Hz (n = 6) in naïve rats. Conversely, all WDR neurons (6/6; 9.3 ± 2.3 Hz) were spontaneously active in inflamed rats (Figure 3B and Figure 4C). In rats with inflammation, SpVc WDR neurons exhibited markedly greater responses to non-noxious mechanical stimuli in comparison to naïve rats (Figure 3B and Figure 4A), as described previously [7,8]. In response to mechanical stimuli (0.2, 6, 15, 60 g), the mean firing rates of SpVc WDR neurons were notably higher in inflamed rats compared to naïve rats (n = 6; Figure 4A) and compared to the 2.1 ± 1.8 g in naïve rats, the mechanical threshold in inflamed rats significantly decreased to 0.6 ± 0.4 g (n = 6; Figure 4B). Inflamed rats exhibited a significantly elevated spontaneous discharge frequency in comparison to naïve rats (Figure 4C). In the inflamed rats, the mean receptive size was substantially higher at 45.5 ± 5.7 mm^2^, in contrast to 22.4 ± 3.8 mm^2^ (n = 6, *p* < 0.05; Figure 4D).

### 3.5. Continuous Administration of NRG Suppress the Excessive Excitability of SpVc WDR Neurons in Inflamed Rats

Utilizing a behavior-based examination to determine the escape threshold, we estimated the impact of prolonged NRG treatment on the hyperexcitability of SpVc WDR neurons in rats with inflammation on day 2. Illustrative examples of SpVc WDR neuron response rates to both non-noxious (0.6–10 g) and noxious (15–60 g) mechanical stimulation following NRG administration are shown in Figure 3C. After two days of daily NRG administration in rats with inflammation, the firing rate of SpVc WDR neurons, which was elevated due to both non-noxious and noxious mechanical stimulation, returned to baseline levels (Figure 3C). The reduced mechanical threshold and increased spontaneous, noxious, and non-noxious firing frequencies in inflamed rats recovered to the levels observed in naïve rats. Following NRG treatment, the typical firing rate of SpVc WDR neurons in inflamed rats showed a significant reduction for both non-noxious and noxious mechanical stimuli (*p* < 0.05, Figure 4A). Following the administration of NRG, the average response to mechanical stimulation in inflamed rats reverted to the levels observed in the naïve control group (Figure 4B). The mean spontaneous firing of SpVc WDR neurons in inflamed rats was also significantly reduced following NRG administration (*p* < 0.05, Figure 4C). The mean receptive field size in rats with inflammation diminished to the levels observed in the naïve group (Figure 4D). Repeated vehicle administration showed no notable impact on the spontaneous or non-noxious, noxious mechanical stimulation-induced hyperactivity of SpVc WDR neurons, consistent with our previous studies [7,8].

### 3.6. Continuous Administration of DIC Reduces the Heightened Excitability of SpVc WDR Neurons in Rats with Inflammation

Next, we examined how chronic administration of DIC influences the hyperexcitability of SpVc WDR neurons in rats with inflammation on day 2. The examples demonstrating how neuron firing rates respond to both non-noxious and noxious mechanical stimuli following DIC administration in rats with inflammation are shown in Figure 3D. The discharge frequency of SpVc WDR neurons to both non-noxious and noxious mechanical stimulation in inflamed rats dropped to control levels after two days of daily DIC administration (Figure 3D). In rats with inflammation, the lowered mechanical threshold and heightened spontaneous, noxious, and non-noxious firing rates were restored to their normal levels after DIC administration (*p* < 0.05). After DIC, the average mechanical stimulation threshold in inflamed rats returned to the levels seen in the naïve group (Figure 4B). In rats with inflammation, the spontaneous activity of SpVc WDR neurons significantly reduced following DIC administration (Figure 4C, *p* < 0.05). The average receptive field size in rats with inflammation was reduced to the levels observed in the naïve control group (Figure 4D).

### 3.7. Chronic Administration of 1/2 DIC + 1/2 NRG Inhibits Hyperexcitability of SpVc WDR Neurons in Inflamed Rats

Finally, our study analyzed the influence of chronic dosing with 1/2 DIC and 1/2 NRG on the hyperexcitability of SpVc WDR neurons in rats that were in their second day of inflammation. Common firing rate patterns observed in SpVc WDR neurons in response to both non-noxious and noxious mechanical stimuli following 1/2 DIC + 1/2 NRG administration in inflamed rats are shown in Figure 3E. The reduced mechanical threshold and increased spontaneous, as well as noxious and non-noxious firing frequencies, in inflamed rats reverted to levels seen in untreated controls. As shown in Figure 4A, there was a significant reduction in the average discharge frequency of SpVc WDR neurons in inflamed rats following the treatment of 1/2 DIC + 1/2 NRG for both non-noxious and noxious mechanical stimuli (*p* < 0.05). The mean mechanical stimulation threshold in inflamed rats following half a dose of DIC and NRG also returned to normal levels (Figure 4B). Administration of 1/2 DIC + 1/2 NRG led to a marked reduction in the spontaneous firing rate of SpVc WDR neurons in inflamed rats (Figure 4C, *p* < 0.05). Rats with inflammation exhibited a significant reduction in mean receptive field size, reaching control levels (Figure 4D).

## 4. Discussion

### 4.1. Administration of NRG Attenuates Trigeminal Inflammatory Hyperalgesia

The objective of this study was to determine if the systemic administration of NRG could alleviate the hyperexcitability of SpVc neurons caused by inflammation and associated with mechanical hyperalgesia. In this research, we discovered the following results: (i) in CFA-inflamed rats, the threshold for escape from mechanical stimulation applied to the orofacial region is significantly lower than that in naïve rats, consistent with previous reports [7,8]; (ii) a reversal of the reduced mechanical threshold to control levels in CFA-inflamed rats on day 2 of systemic administration of NRG; and (iii) on day 2, the vehicle administration had no significant impact on the escape threshold in rats with CFA-induced inflammation. Previous studies reported that administration of 50 mg/kg NRG inhibited CFA-induced inflammatory nociceptive behavior and reduced PGE_2_-induced mechanical hyperalgesia in both in vitro and in vivo conditions [10,16]. The findings from this study are consistent with earlier research, which demonstrated that dietary components, such as polyphenols and carotenoids, could significantly decrease CFA inflammation-induced mechanical hyperalgesia in a rat model of inflammatory pain [7,8]. Taken together, the results imply that a daily dose of NRG alleviates the inflammation-driven hypersensitivity in the whisker pads of rats through the suppression of Cox-2 signaling pathways, leading to the suppression of PGE2 production via previously described mechanisms [7].

### 4.2. NRG Alleviates the Hyperexcitability of SpVc WDR Neuron Activity Connected with Hyperalgesia Post-Inflammation

The commonly acknowledged process for nociceptive sensory signaling relies on four main steps: initial transduction at peripheral terminals to convert external stimuli; subsequent generation of action potentials; propagation of action potentials along axons; and, finally, transmission to central terminals that serve as the presynaptic elements of the initial synapses in the sensory pathways of the central nervous system [2,24].

Concerning the generator potential associated with TRP channels, it has been reported that NRG inhibits the current of TRPM3 in DRG neurons [14]. TRPM3 is expressed in nociceptive sensory neurons in the DRG and TG. Since TRPM3^−/−^ mice specifically show an attenuated response to thermal nociceptive stimuli, an effect that is maintained after induction of inflammatory hyperalgesia [25], TRPM3 activation has been linked to thermal pain associated with inflammation. Previously, it has been reported that NRG induces analgesia through activation of the NO/c-GMP/PKG/ATP-sensitive K channel pathway, reducing neuronal pain transmission in inflammatory pain models [10,15]. Neuronal NO synthase generates NO, subsequently triggering the cyclic GMP-PKG-ATP-sensitive K channel pathway at both DRG and spinal cord levels, indicating analgesia. However, to our knowledge, systemic in vivo electrophysiological studies have not been conducted, and the electrophysiological mechanism of how NRG suppresses the hyperexcitability of nociceptive neurons induced by inflammation has not been elucidated.

This study shows that the systemic application of NRG reversed the reduction in the average mechanical stimulation threshold noted in rats with inflammation. This led to the mean discharge frequency of SpVc WDR neurons, triggered by both non-noxious and noxious mechanical inputs, reverting to pre-inflammation levels. PGE_2_, a proinflammatory mediator, binds to G protein-coupled E-type prostanoid (EP) receptors, which in turn can activate protein kinase A in nociceptive peripheral terminals following peripheral inflammation, initiating activation of the signaling pathway [26]. Zhou et al. [13] demonstrated that under in vitro conditions, the application of NRG inhibits tetrodotoxin-resistant (TTX-R) Nav currents in the primary sensory neurons of the DRG. Consequently, our research suggests that systemic NRG might influence the peripheral sensitization caused by inflammation and the hypersensitivity of SpVc WDR neurons in peripheral nerve terminals, as suggested by previous in vitro findings, via modulation of Nav 1.8 TTX-R Nav channels [13]. TTX-R Nav channels seem to be selectively present in nociceptive DRG neurons, which correspond to Aδ/C-primary afferent TG neurons, while TTX-S Nav channels are found in Aβ/Aδ-primary afferent TG neurons [27,28]. A previous study reported that NRG could also decrease the production of PGE_2_ by inhibiting Cox-2 cascades [16], findings that suggest NRG could inhibit inflammation-induced peripheral sensitization of trigeminal primary afferent terminals in inflamed tissues.

Furthermore, the current research demonstrates that NRG counteracted the elevated mean spontaneous discharge frequency of SpVc WDR neurons resulting from inflammation. Previous reports have linked the ongoing actions in the SpVc to enduring headaches (spontaneous pain) [29]. Recent studies have shown that the prolonged activation of WDR neurons in the SpVc is mainly caused by signals from the peripheral region, as evidenced by a significant reduction in continuous activity when lidocaine is administered into the TG [30]. When combined with the current findings, this indicates that NRG reduces the augmented spontaneous discharge activity of SpVc WDR neurons connected with the whisker pad is caused by sensitization occurring at the peripheral and/or trigeminal ganglion level.

Previously, it has been demonstrated that using in vitro spinal cord slice preparations, NRG reduced both the frequency and amplitude of the spontaneous excitatory post-synaptic current, suggesting that NRG inhibits nociceptive neuronal transmission via pre- and post-synaptic inhibitory mechanisms [13]. Since Zhou et al. [13] demonstrated that under in vitro conditions, application of NRG inhibited N-type Cav currents in the primary sensory neurons of the DRG, it can be assumed that application of NRG decreases the release of glutamate from nerve terminals via inhibition of N-type Ca V channels. As a result, the amplitude of excitatory post-synaptic potentials of nociceptive SpVc WDR neurons is reduced, resulting in a decrease in action potential firings.

Our previous research indicated that a local GABAergic mechanism has the potential to modulate nociceptive transmission in SpVc neurons, which in turn influences their general properties of mechanical receptive fields [31]. This study found that the increased receptive field size in rats with inflammation returned to baseline after NRG treatment, even though the exact processes behind this effect are not well understood. A previous study demonstrated that flavonoids, including NRG, which are polyphenolic compounds, have allosteric effects on GABA_A_ receptors and their binding affinity [32,33]. Therefore, NRG may potentially regulate local GABAergic tonic regulation and manage nociceptive and mechanoreceptive signaling while suppressing central mechanisms via excitatory synaptic transmission. Previously, we observed that the size modifications in the mechanical receptive field were a result of the local iontophoretic use of GABA_A_ receptor agonists and antagonists [31]. It is possible to assume that the application of NRG leads to a reduction in receptive field sizes through the modulation (activation) of GABAergic inhibitory mechanisms in the SpVc local circuit. Nevertheless, further examinations are required to substantiate this hypothesis.

Currently, it was observed that injury to the trigeminal nerve or inflammation of surrounding tissues might cause increased excitability in trigeminal primary afferent neurons, leading to activation of microglia in the trigeminal brainstem sensory nuclear complex and accumulation of macrophages [7,22,34]. The interactions among satellite glial cells, macrophages, and hyperactivated TG neurons are facilitated by a range of mediators and receptor mechanisms, and the communication between satellite glial cells, macrophages, and hyperactive TG neurons is enabled by various mediators, receptor mechanisms, and signaling pathways [7,22,34]. Through this form of communication, there might be an additional increase in the excitability of TG afferent neurons, evidenced by their heightened response in input components of the SpVc neurons. The initiation of SpVc triggers the release of mediators that induce hyperactivity in neurons, leading to the activation of microglia and astrocytes. Communication between neurons and non-neuronal cells through different molecules plays an important role in preserving the hyperresponsive state of neurons (central sensitization) [7,22,34]. While these findings indicate that NRG may alter the neuronal hyperexcitability of SpVc neurons by means of various molecules, to the best of our knowledge, there are no documented reports on the effect of NRG on the excitability of nociceptive neurons via neuron–glial interaction, and additional research is needed to evaluate this possibility.

To our knowledge, studies have reported the impact of NRG on pain sensitivity across different sexes [13]. Previous research noted variations in experimental pain sensitivity across different phases of the menstrual cycle, demonstrating that sensitivity to several kinds of pain is greater in the luteal phase than in the follicular phase [35]. While it is commonly acknowledged that sex hormones play a major role in pain variability, the detailed reasons for the disparity are still unclear [21]. Consequently, our hypothesis has been tested solely on male rats, and additional research is required to clarify any gender-related variations.

### 4.3. The Importance of NRG in Suppressing the Hyperactive Response of SpVc Neurons in Connection to Pain Hypersensitivity

When conventional medical treatments are unsuccessful, CAM therapies like herbal remedies and acupuncture are often adopted for controlling pain [6]. Continuous ingestion of dietary substances, like polyphenols and carotenoids, has been found to decrease mechanical hypersensitivity caused by inflammation, mainly by reducing the hyperactivity of SpVc WDR neurons through both peripheral and central Cox-2 cascade signaling routes [7,8]. In the human diet, flavonoids represent the most prevalent category of polyphenolic compounds obtained from plants, notably flavanones like NRG, which is a group of polyphenols found predominantly in citrus fruits.

In this study, we made the following observations: (i) restoration of the lowered mechanical threshold to normal levels in inflamed rats by the second day of consistent DIC treatment or a combination of half DIC and half NRG treatment; (ii) in inflamed rats, the average firing rate of SpVc WDR neurons in response to both non-noxious and noxious mechanical stimuli was notably reduced following the treatment of DIC or 1/2 DIC + 1/2 NRG administration; (iii) the heightened average spontaneous activity of SpVc WDR in rats with inflammation was notably reduced following DIC treatment or a combination of half DIC and half NRG administration; and (iv) treatment with DIC or a mix of half DIC and half NRG normalized the enlarged mean receptive field size in rats with inflammation. In this research, the extent of NRG-mediated suppression of SpVc neuron hyperexcitability linked to hyperalgesia was nearly equivalent to that of DIC (50 mg/kg, i.p.), implying NRG is equivalent to DIC and has potential as a therapeutic agent in CAM strategies for the prevention of trigeminal inflammatory mechanical hyperalgesia. Furthermore, this idea was supported by evidence that the extent of NRG-mediated suppression of the hyperexcitability in SpVc neurons related to hyperalgesia was nearly equivalent to a combination of half DIC and half NRG.

In regard to orofacial pain, it is generally understood in clinical settings that the majority of patients going through orthodontic therapy report experiencing pain, including referred pain, and NSAIDs are commonly prescribed to manage pain symptoms. Orthodontic appliances apply mechanical forces to bring about tooth movement. In the process of orthodontic treatment, inflammation of the gums and bone absorption on the side experiencing tension are commonly noted [36]. Due to the significant role of PGE_2_ in osteoclast-related bone remodeling, NSAIDs are known to have detrimental effects on orthodontic patients, including decreased tooth movement [37,38]. Recently, it has been reported that the prolonged intake of the phytochemical resveratrol can lessen the mechanical, ectopic hyperalgesia induced by tooth movement experiments, which is associated with hyperexcitable SpVc WDR neurons in anesthetized rats [39]. These findings indicate that this dietary component could serve as a promising treatment for ectopic pain, such as the pain experienced by individuals undergoing orthodontic treatment [39]. In the present study, we found that systemic administration of a half-dose of DIC replaced the half-dose of NRG. These results indicate that administering NRG might reduce the unwanted hyperalgesia caused by orthodontic treatment without causing any side effects. Nonetheless, additional research is necessary to clarify this possibility.

**Figure 5:**
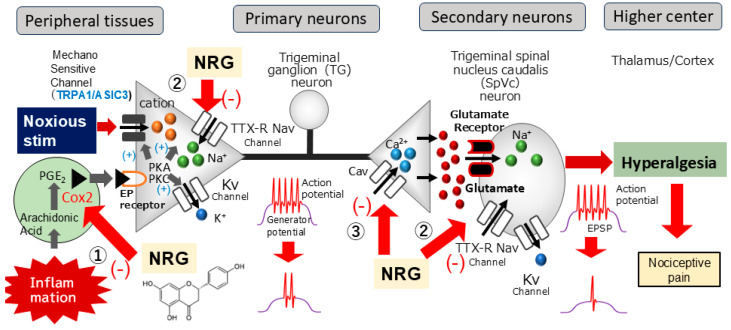
A possible mechanism underlying NRG suppression of inflammation-induced mechanical hyperalgesia. Following peripheral inflammation, inflammatory mediators, such as prostaglandins (PGE_2_), attach to G protein-coupled E prostanoid (EP) receptors, triggering the activation of protein kinase A and C (PKA and PKC, respectively) in pain-detecting peripheral nerve endings, leading to phosphorylation of mechanosensitive transient receptor potential/acid-sensing ion channels (TRP/ASIC), Na^+^ (Nav), and K^+^ (Kv) channels and receptors. As a result, the activation threshold for transducer channels, such as the TRP channel family, including TRPA1, is diminished, leading to an increase in membrane excitability at the peripheral terminals, which causes a higher frequency of action potentials being conducted to presynaptic central terminals of the SpVc. Ultimately, more glutamate is released into the synaptic cleft and binds to the increased number of post-synaptic glutamate receptors, augmenting excitatory post-synaptic potentials (EPSPs) and triggering a flow of action potentials transmitted to higher pain pathway centers, producing an intensified sensitivity referred to as peripheral sensitization. There is a possibility that systemic delivery of NRG reduces hyperalgesia caused by inflammation-induced mechanical hyperalgesia, with this effect primarily due to suppression of the hyperexcitability of SpVc WDR neurons via inhibition of the peripheral cyclooxygenase (Cox)-2 cascade signaling pathways (①), tetrodotoxin-resistant (TTX-R) Nav channels (②), and the central terminal of Cav channels (③), decreasing the firing frequency of action potentials in the nociceptive nerve terminals and inhibiting the conduction of pain signals to the SpVc and higher centers for lateral and medial pain control (hyperalgesia).

This research is the first to analyze the suppressive capacity of NRG and the NSAID DIC in relation to inflammation-triggered SpVc neuronal excitability associated with hyperalgesia. As summarized in Figure 5, with systemic administration of NRG, it is possible that NRG alleviates mechanical inflammatory hyperalgesia caused by inflammation, chiefly through the inhibition of hyperexcitability of SpVc WDR neurons through the blockade of the peripheral Cox-2 cascade signaling pathways, TTX-R Nav channels, and the central terminal of Cav channels, decreasing the firing frequency of action potentials in the nociceptive nerve terminals and inhibiting the conduction of pain signals to the SpVc and higher centers (hyperalgesia). Consequently, these outcomes play a role in advancing the formulation of analgesic drugs designed to address and prevent trigeminal inflammatory pathological pain, including clinical orofacial pain, with fewer side effects. These results also suggest the possibility of treating pain in a natural way through the intake of certain dietary and nutritional ingredients that contain naringenin.

## 5. Conclusions

In the present study, we demonstrate for the first time that systemic administration of the phytochemical NRG can mitigate inflammatory mechanical hyperalgesia related to the increased excitability of nociceptive SpVc WDR neurons by blocking the Cox-2 signaling pathway. The data support the idea that NRG could be a viable therapeutic option instead of NSAIDs in CAM methods to prevent trigeminal inflammatory mechanical hyperalgesia. This idea requires further research to determine whether NRG is involved in the relief of clinical pain, such as orthodontic pain, and whether it has the same effect as specific Cox-2 inhibitors.

## Data Availability

All data from this study are included in the main body of the article.
